# Preservation and clonal behavior of extrachromosomal DNA in patient-derived xenograft models of childhood cancers

**DOI:** 10.1186/s13073-026-01676-0

**Published:** 2026-05-28

**Authors:** Rishaan Kenkre, Owen S. Chapman, Eugene Yui-Ching Chow, Jens Luebeck, Yan Yuen Lo, Megan Paul, Wenshu Zhang, Jill Mesirov, Vineet Bafna, Kevin Yip, Jon D. Larson, Robert J. Wechsler-Reya, Lukas Chavez

**Affiliations:** 1 Sanford Burnham Prebys Medical Discovery Institute, 10901 North Torrey Pines Road, La Jolla, San Diego, CA 92037 USA; 2 Department of Neuro-Oncology, Institute of Brain Sciences, Nagoya City University Graduate School of Medical Sciences, Nagoya, Japan; 3 Department of Computer Science and Engineering, University of California San Diego, La Jolla, San Diego, CA USA; 4 Rady Children’s Hospital San Diego, San Diego, CA USA; 5 Department of Pediatrics, University of California San Diego, La Jolla, San Diego, CA USA; 6 Bioinformatics and Systems Biology Graduate Program, University of California San Diego, La Jolla, San Diego, CA USA; 7 School of Medicine, University of California San Diego, La Jolla, San Diego, CA USA; 8 Moores Cancer Center, University of California San Diego, San Diego, CA USA; 9 Halicioglu Data Science Institute, University of California San Diego, San Diego, CA USA; 10 Department of Neurology and Herbert Irving Comprehensive Cancer Center, Columbia University Irving Medical Center, New York, NY USA; 11 Department of Medicine, University of California San Diego, La Jolla, San Diego, CA USA; 12 Rady Children’s Institute for Genomic Medicine, Rady Children’s Hospital and Healthcare Center, San Diego, CA USA

**Keywords:** Extrachromosomal DNA, Pediatric cancer, Patient-derived xenografts, Medulloblastoma, Intratumoral heterogeneity

## Abstract

**Background:**

Extrachromosomal DNA (ecDNA) is a structural variant linked to poor prognosis in pediatric cancers. Patient-derived xenograft (PDX) models are crucial tools for cancer research, as they are believed to recapitulate the molecular features and intratumoral heterogeneity in patient tumors. However, ecDNA demonstrates unique evolutionary dynamics under selective pressure, and its behavior during PDX development remains largely uncharacterized. This study investigates the fidelity of PDX models in representing ecDNA from primary tumors. By analyzing ecDNA sequence composition and copy number conservation across pediatric solid cancers, we assess how well PDX models recapitulate the ecDNA landscape observed in human tumors.

**Methods:**

AmpliconArchitect was used to analyze whole-genome sequencing (WGS) of 338 PDX models and 127 corresponding primary tumors. ecDNA status, sequence, copy number, and associated genes were compared between PDX models and their matched human tumors. Additionally, multiome RNA and ATAC single-cell sequencing of a PDX tumor enabled comparison of ecDNA intratumoral heterogeneity relative to similar data from the primary tumor.

**Results:**

ecDNA in PDX models largely recapitulated oncogene amplifications observed in human tumors, with *MYCN* being the most frequently amplified. ecDNA status remained unchanged for a majority of the PDX models (105/127, 83%) compared to primary tumors, with 20% of previously ecDNA-negative cases acquiring ecDNA during PDX development. Consequently, ecDNA was more prevalent in the PDX models than in their corresponding human tumors (McNemar's test, *p* = 0.00086). Detailed examination of ecDNA sequences in tumor-PDX pairs showed substantial conservation (67% with > 90% sequence overlap) but variable breakpoint concordance. Single-cell analysis demonstrated that rare ecDNA-positive cells from the primary tumor preferentially drive PDX tumor development.

**Conclusion:**

This study highlights the prevalence, oncogenic content, and conservation of ecDNA in PDX models relative to pediatric patient tumors. We observed that ecDNA frequently recapitulates oncogene amplifications found in human cancers, is generally preserved during PDX establishment, and reflects subtype-specific patterns across tumor types. These findings support the utility of PDX models in studying ecDNA biology in pediatric cancer progression and therapy. Longitudinal sampling during PDX tumor growth and under therapeutic pressure could provide insights into molecular evolution, clonal selection, and ecDNA-driven therapy resistance.

**Supplementary Information:**

The online version contains supplementary material available at 10.1186/s13073-026-01676-0.

## Background

Extrachromosomal DNA (ecDNA), also known as double minutes, is a critical oncogenic driver in many different types of cancer [1–4]. Childhood cancer patients whose tumors contain ecDNA have significantly worse 5-year survival than patients whose tumors have chromosomal or no amplifications [3–5]. Due to the absence of centromeres, ecDNA exhibits random inheritance, driving copy number amplification, promoting intratumoral heterogeneity, accelerating tumor evolution, and contributing to treatment resistance [6]. These features emphasize the importance of studying the role of ecDNA during tumor development, recurrence, and evolution.

**Fig. 1:**
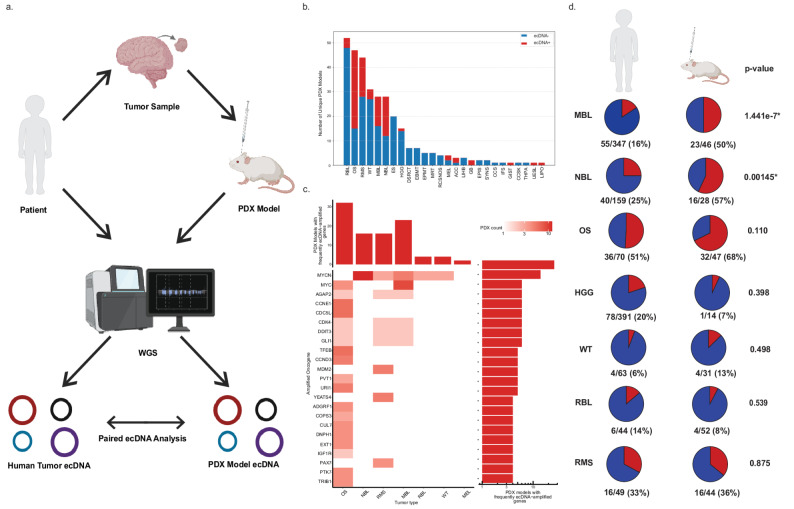
Landscape of ecDNA in pediatric cancer patient-derived xenograft models. **a** Graphical abstract of the study design displaying each stage from tumor sample extraction to paired ecDNA analysis. **b** Distribution of PDX models across childhood cancer types. Blue bars show the number of ecDNA-negative PDX models by cancer type. Red bars show the number of ecDNA-positive PDX models by cancer type. **c** Heatmap indicates the number of PDX samples with ecDNA amplification of a given recurrently amplified gene (n > 3) in that tumor type. The top left barplot indicates the number of PDX models with ecDNA sequences amplifying these genes for each cancer type. The right barplot demonstrates the number of ecDNA sequences amplifying the indicated gene across all tumor types. **d** Pie charts displaying ecDNA prevalence in primary human tumors of different childhood cancer types as analyzed in a previous pediatric pan cancer study of ecDNA (left column) compared to PDX models analyzed in this study (right column). Red = ecDNA-positive, blue = ecDNA-negative. Statistical analysis shows significant enrichment of ecDNA prevalence in PDX tumors for medulloblastoma and neuroblastoma (chi-squared test of independence p-values shown for each tumor type in the right columns). Similarly, an increased frequency of ecDNA was observed in PDX models derived from osteosarcoma patients; however, enrichment was below significance

Patient-derived models, such as cell lines and orthotopic xenograft (PDX) mouse models, are widely used to study tumor biology and therapeutic vulnerabilities. Pharmacological inhibition studies in PDX mouse models are important preclinical experiments for evaluating tumor responses in vivo. PDX models largely recapitulate the histological features, DNA methylation profiles, and intratumoral heterogeneity of the tumors from which they were derived [7, 8]. However, the behavior of ecDNA during PDX model development and propagation has not yet been analyzed. To evaluate the representation of ecDNA in PDX models compared to the tumors from which the models were derived, this study analyzes the occurrence and preservation of ecDNA sequences and copy numbers in PDX models compared to human tumors in a broad spectrum of childhood solid cancers (Fig. 1a). To facilitate investigation of the PDX samples of interest, the dataset is available through an interactive web portal at https://ccdi-ecdna.org/pdx.

## Methods

### Study cohort and sample description

WGS data of PDX samples and patient tumors was accessed using the St Jude Cloud Data Portal (https://platform.stjude.cloud/data/) [9]. In addition, low-coverage WGS data for MBL PDX models and human tumors were obtained from a previous publication [10]. Eligibility for inclusion was determined by the availability of samples in the respective data portals. No attrition was recorded during the study period.

The full dataset comprised 338 PDX samples derived from 288 patients across 31 childhood cancer types. In some cases, multiple PDX models were descended from the same patient tumor, leading to more than one PDX sample per patient in the dataset. For paired ecDNA status and sequence analyses, a subset of 127 samples with WGS data available for both the primary human tumor and its corresponding PDX model were analyzed. Copy number analyses from the AmpliconSuite results were performed on 23 primary tumors with matched PDX models in which ecDNA-amplified genes were confirmed in both samples and amplicon overlap coverage was at least 90%. Pairwise ecDNA sequence similarity analyses were performed on the 30 tumor-PDX pairs in which both the human tumor and PDX model were ecDNA-positive. For single-cell analyses, multiome ATAC and RNA sequencing data were generated from RCMB56-pdx and RC124-pdx as described below, and compared to previously published data from RCMB56-ht [3]. A full description of all samples and associated metadata is provided in Additional file 1: Table S1.

### Whole genome sequencing (WGS) of PDX samples and patient tumors processing

WGS data was preprocessed and aligned according to the internal pipelines at St. Jude (hg38). Docker containers of Amplicon Architect software were installed on the DNANexus cloud genomics platform (see Methods: “ecDNA detection and classification”).

### ecDNA detection and classification from bulk WGS

To detect ecDNA, all samples in the WGS cohort were analyzed using the AmpliconSuite-pipeline [11] v1.1.0, AmpliconArchitect [12] v1.3, and AmpliconClassifier [13] v0.4.4, either with the GRCh38 or GRCh37 reference genomes. Briefly, the Amplicon Architect algorithm was performed as follows. Copy number segmentation and estimation were performed using the CNVkit v0.9.664 [14]. Segments with copy number = 4.5 were extracted using AmpliconSuite-Pipeline (November 2023 update) as “seed” regions. For each seed, AmpliconArchitect searches for the region and nearby loci for discordant read pairs that are indicative of genomic structural rearrangements. Genomic segments are defined based on boundaries formed by genomic breakpoint locations (identified by discordant reads) and modulations in genomic copy number. A breakpoint graph of the amplicon region was constructed using CN-aware segments, and genomic breakpoints and cyclic paths were extracted from the graph. Amplicons were classified as ecDNA, breakage-fusion-bridge, complex, linear, or no focal amplification using the heuristic-based companion script AmpliconClassifier. Biological samples with one or more classifications of “ecDNA” were considered ecDNA-positive, and all others were considered ecDNA-negative.

Code is available at:

- AmpliconSuite pipeline: https://github.com/AmpliconSuite/AmpliconSuite-pipeline [11]
- PrepareAA: https://github.com/jluebeck/PrepareAA [12]
- AmpliconArchitect: https://github.com/virajbdeshpande/AmpliconArchitect [12]
- AmpliconClassifier: https://github.com/jluebeck/AmpliconClassifier [11]
- CycleViz: https://github.com/AmpliconSuite/CycleViz/ [11]
- DNANexus AmpliconSuite https://github.com/chavez-lab/ampliconsuite-dnanexus-applet [15]

### Patient metadata, survival, and Medulloblastoma (MBL) subgroup annotation

Where available, patient samples and models were assigned metadata annotations including age, sex, survival, and MBL subgroup, based on previously published annotations of the same tumor or model [10, 16–21]. Sample metadata are also available in some cases from the respective cloud genomics data platforms: https://dcc.icgc.org/ (ICGC) [22], https://pedcbioportal.kidsfirstdrc.org/ [23], https://portal.kidsfirstdrc.org/ (CBTN) [24], and https://pecan.stjude.cloud/ (St Jude) [9]. Where primary sources disagreed on a metadata value, that value was reassigned to the NA. Patient tumors from the CBTN were assigned molecular subgroups based on the consensus of two molecular classifiers using RSEM-normalized FPKM data: MM2S69 and D3b medulloblastoma classifier at the Children’s Hospital of Philadelphia (https://github.com/d3b-center/medullo-classifier-package) [25]. To determine the molecular subgroup of PDX samples, we generated or obtained DNA methylation profiles (Illumina 450 k or EPIC) from a previous publication [26] and classified samples by molecular subgroup according to the DKFZ brain tumor methylation classifier (https://www.molecularneuropathology.org/mnp) [26].

### Establishment and maintenance of RCMB56-pdx and RC124-pdx

RCMB56-pdx and RC124-pdx were established by implanting 0.5–1 × 106 dissociated patient tumor cells directly into the cerebellum of NSG mice. Subsequent tumors were harvested from the mice, dissociated, and re-implanted into new NSG mice without in vitro passage. Ex vivo experiments were performed using RCMB56-pdx and RC124-pdx cells at passage 1 (p1) or higher.

### Multiome single-cell seq

We generated new multiome single-cell sequencing data from the corresponding PDX model RCMB56-pdx as previously described [3]. Briefly, fresh PDX tumor cells were dissociated for single-cell multiome ATAC and gene expression sequencing (10X) according to the manufacturer’s instructions. Sequencing was performed using an Illumina NovaSeq S4 200 instrument at a depth of at least 250 M reads for scATAC-seq and 200 M reads for scRNA-seq. In addition, multiome single-cell sequencing data of the human tumor RCMB56-ht was made available by Chapman et al. [3].

Additionally, multiome single-cell sequencing was performed in the same manner on the primary G3 MBL tumor RC124-ht and its corresponding PDX model RC124-pdx.

### Pairwise similarity of ecDNAs sequences

Overlapping focal amplifications were compared to assess amplicon similarity based on the relative degrees of shared overlap in genomic coordinates and structural variant (SV) breakpoint locations. These calculations were implemented in the feature_similarity.py script, available in the AmpliconClassifier repository (https://github.com/AmpliconSuite/AmpliconClassifier) [11].

We defined two measurements of similarity based on Jaccard indices. JaccardGenomicSegment similarity is a Jaccard index computed using two sets formed by the coordinate ranges of genomic intervals comprising two focal amplifications. The second is Jaccard–breakpoint similarity, which is a Jaccard index computed on two sets formed by the locations of SV breakpoint junctions in the two focal amplifications. Two SV breakpoint junctions were determined to be the same if the total absolute difference between the measured genomic endpoints of the junction was less than 250 bp [13].

The amplicon feature similarity script (https://github.com/AmpliconSuite/AmpliconClassifier/blob/main/feature_similarity.py) [11] supports the comparison of amplicons globally for all amplicon regions, but can also be run in a restricted mode, which limits the comparison to specific regions of the genome. Furthermore, as AmpliconArchitect may include flanking regions that are not focally amplified as part of the amplification itself, the amplicon similarity script filters from the calculation regions that are not focally amplified (CN < 4.5 default), as well as redundant filter regions that are also present in the low-complexity or low-mappability database used by AmpliconArchitect.

In addition to the metrics output by the amplicon feature similarity script, we also calculated the amplicon length coverage percentage. This metric was computed as $\frac{\mathrm{A}\mathrm{m}\mathrm{p}\mathrm{O}\mathrm{v}\mathrm{e}\mathrm{r}\mathrm{l}\mathrm{a}\mathrm{p}\mathrm{L}\mathrm{e}\mathrm{n}}{\mathrm{min}\left(Amp1AmpLen,Amp2AmpLen\right)}*100$ to determine how well the amplicons overlap between a pair covering the amplicon with the lower length.

### Statistical methods

Statistical tests, test statistics, and p-values are indicated where appropriate in the main text. Categorical associations were established using the chi-square test of independence [27] if N > 5 for all categories; otherwise, the Fisher’s exact test [28] was used. For both tests, the Python package scipy.stats v1.5.3 implementation was used [29]. In addition, statistical analysis for the paired tumors was performed using the McNemar test [30] implemented in statsmodels v0.12.0 [31].

### Single cell data processing and clustering

Sequencing data for both tumor-PDX pairs (RCMB56-ht/pdx and RC124-ht/pdx) were uniformly processed using CellRanger ARC (v2.0.0 for RCMB56, v2.0.2 for RC124) [32] with default parameters, followed by Seurat (v4.0.4 for RCMB56, v5.3.0 for RC124) [33]. For RCMB56, cell barcodes were retained based on quality thresholds: ATAC mitochondrial fraction < 0.1, ATAC read count between 1,000–70,000, and RNA read count between 500 (ht) or 1000 (pdx) and 25,000. Doublets were identified and removed using DoubletFinder v2.0 (RRID:SCR_018771) [34] with default parameters. Host cells were removed from PDX samples using Xenocell v1.0.1 [35], filtering barcodes with < 90% uniquely mapped reads to the human genome. Following preprocessing, 2,986 RCMB56-ht and 10,400 RCMB56-pdx cells remained, whereas 5,669 RC124-ht and 6,338 RC124-pdx cells were present after applying similar filtering criteria.

Clustering was performed independently for each sample using the weighted nearest neighbor algorithm [36] with default parameters. Cell-type identities were assigned by identifying differentially expressed genes (DEGs) for each cluster using Seurat's FindAllMarkers function and cross-referencing against known cell type marker genes [37]. Only primary tumor clusters (RCMB56-ht and RC124-ht) were labeled in this manner. For RC124 copy number analysis, ecDNA-quant outputs (see below) were additionally utilized for cell type identification.

### Identification of ecDNA-containing cells

ecDNA-containing cells were identified by permutation tests comparing scATAC-seq read coverage at the ecDNA locus, using AmpliconArchitect on bulk WGS, as described above, to read coverage elsewhere in the genome. This code is available at https://github.com/auberginekenobi/ecdna-quant [3]. Briefly, deduplicated scATAC-seq reads were obtained from the fragments.tsv output of CellRanger ARC and sorted by barcode. For Monte Carlo permutation testing [38], 1000 random contiguous regions of the genome, excluding centromeres, telomeres, known ecDNAs, and low-mappability regions, were generated using bedtools v2.27.1 [39]. Read coverage was counted using PyRanges v0.0.112 [40] and scaled to region length. For each cell, empirical *p*-values were estimated as *p*^ = (*r* + 1)/(*n* + 1) [41]. Multiple hypothesis correction was performed using the Benjamini–Hochberg correction [42]. Z-scores were calculated using the standard formula, comparing the average read coverage at the ecDNA-amplified region to the mean and variance of the Monte Carlo permutations.

### Single-cell genomic copy number estimation

To investigate copy number aberrations based on single-nucleus ATAC-sequencing data, we used an adapted version of InferCNV as described in Okonechnikov et al. [43].

### Animals

NOD-SCID IL2Rγ null (NSG) mice (Jackson Laboratory, strain no. 005557) were housed in an aseptic barrier research animal facility at the Sanford Consortium for Regenerative Medicine, with a 12 h light–dark cycle, ambient temperature of 19–24 °C and 40–60% humidity. All experiments were performed in accordance with national guidelines and regulations, and according to protocols approved by the Animal Care and Use Committees at the Sanford Burnham Prebys Medical Discovery Institute and UCSD (San Diego, CA, USA) and the UCSD Institutional Review Board (Project number 190055). In compliance with humane endpoint protocols, tumor-bearing mice displaying signs of moribundity (dysmorphic head, hunched posture, ataxia, excessive weight loss) were euthanized and processed without exceeding tumor burden limitations.

## Results

### ecDNA in human tumors and PDX models amplify the same oncogenes

To investigate ecDNA in PDX models of childhood cancers, we accessed whole-genome sequencing (WGS) data from St. Jude Cloud’s Childhood Solid Tumor Network [10, 44], a medulloblastoma (MBL) cohort from Rady’s Children’s Hospital, San Diego, and other sources [3] (Additional file 1: Table S1). By employing AmpliconArchitect (AA) [12] and AmpliconClassifier (AC) [13], we identified 175 ecDNA sequences in 106 PDX models (31%) derived from 93 patients across 14 cancer types (Fig. 1b, Additional file 2: Table S2). When compared to a reference cohort of childhood cancer patients [5], we observe a statistically significant increase of ecDNA in this cohort of PDX tumors (8.8% vs. 31.4% ecDNA-positive human tumors or PDX models, respectively, Fisher's exact test, *p* < 0.001).

In this cohort of 106 ecDNA-positive PDX models, a total of 23 different oncogenes were recurrently (*n* > 3) amplified on ecDNA (Fig. 1c). As in primary childhood cancers [5], *MYCN* was the most frequently amplified oncogene with occurrence in 24 PDX models derived from neuroblastoma (NBL), rhabdomyosarcoma (RMS), Wilms tumor (WT), retinoblastoma (RBL), and MBL [13–15] Furthermore, *MYC*, *CCNE1*, *MDM2*, and *IGF1R*, showed consistent amplification patterns in both PDX models and human tumors, with *MYC* most prevalent in MBL, *CCNE1* and *IGF1R* most prevalent in OST, and *MDM2* most prevalent in RMS (Fig. 1c). Notably, *CUL7* amplification (*n* = 4 PDX models) was detected exclusively in the PDX cohort but not in the reference human tumor population of childhood cancer patients (*N* = 3,631) [5]. *CUL7* serves a role as both a tumor promoter and suppressor, influencing tumor growth and development in various cancers, mainly through its effect on substrate stability and thus cell signaling pathway regulation [45, 46]. Overall, these analyses demonstrate that ecDNA in PDX models of childhood cancers largely recapitulates the oncogene amplifications observed in human tumors. The persistent amplification of key oncogenes such as *MYCN* and *MDM2* across both PDX models and primary tumors validates the biological relevance of these models in investigating ecDNA-driven oncogenesis.

### ecDNA amplifications are enriched in PDX models

To evaluate the frequency of ecDNA in PDX models compared to the reference cohort of primary human tumors, we analyzed seven pediatric cancer types for which at least five PDX models were available (262 PDX models in total). Statistical analysis revealed enrichment of ecDNA detected in PDX compared to the reference human tumors in MBL and NBL (?2 = 27.7 and 10.1, *p* = 1.4e-7 and 0.0015 respectively, Fig. 1d). A similar but nonsignificant trend was observed in OST tumors (chi-squared test of independence, *p* = 0.110). For the remaining tumor types, no difference in the frequency of ecDNA was observed compared to PDX models and human tumors (Fig. 1d).

**Fig. 2:**
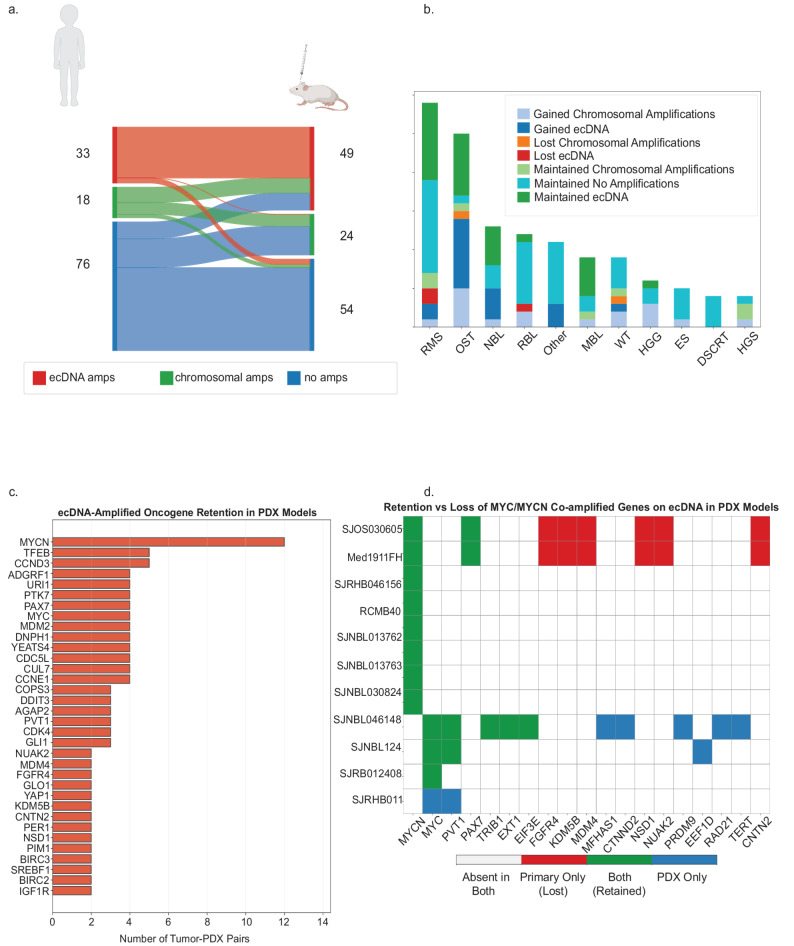
ecDNA amplification status and oncogene retention in human tumors and PDX models. **a** Sankey diagram that displays the flow of ecDNA status from human tumors to the corresponding PDX models for 127 pairs. Primary tumors on the left side and PDX models on the right side. Blue flows indicate primary tumor is ecDNA-negative with no other amplifications; green flows indicate primary tumor is ecDNA-negative with chromosomal amplifications; red flows indicate primary sample is ecDNA-positive. **b** Stacked bar chart demonstrating transitions of focal amplification status across human tumor-PDX pairs grouped by cancer type. [RMS = Rhabdomyosarcoma, OST = Osteosarcoma, NBL = Neuroblastoma, RBL = Retinoblastoma, Other = Low-incidence tumor types (n<3), MBL = Medulloblastoma, WT = Wilms' Tumor, HGG = High-Grade Glioma, ES = Ewing Sarcoma, DSCRT = Desmoplastic Small Round Cell Tumor, HGS = High Grade Sarcoma] (**c**) Horizontal bar chart showing ecDNA-amplified oncogene retention across tumor-PDX pairs, ranked by sample size. All 33 oncogenes analyzed demonstrated 100% retention in PDX models. Fisher's exact test with FDR correction applied across all such ecDNA-amplified oncogenes. MYCN showed the strongest association with ecDNA retention (n = 12 pairs, FDR = 0.00015). **d** Gene-sample matrix heatmap of co-amplified genes with MYC or MYCN on ecDNA, along with whether they are coordinately retained or independently lost in PDX models

Enrichment of ecDNA in PDX models compared to the patient reference cohort is challenging to interpret due to potential differences in sampling methodology and tumor selection criteria between the two cohorts. To enable a direct comparison, we next focused our analysis on 127 patients for whom WGS data are available for both the primary human tumors and the PDX models derived from these tumors (Additional file 3: Table S3). In the majority of cases (105/127, 82.68%), ecDNA status remained unchanged in the PDX models compared to the primary tumors (Fig. 2a). This included 30 out of 33 patients (90.9%) whose tumors remained ecDNA-positive, with only 3/33 (9.09%) cases losing ecDNA in PDX models. Notably, 19/94 (20.21%) ecDNA-negative primary tumors gained ecDNA in their corresponding PDX models (Fig. 2a, Additional file 3: Table S3). Consequently, ecDNA was more prevalent in the PDX models than in their corresponding human tumors (McNemar’s test, *p* = 0.00086). Among the 94 ecDNA-negative primary tumors, two distinct patterns emerged based on their amplification status. For the 18 tumors with chromosomal amplifications, half (50%) of their PDX models converted to ecDNA amplifications, while 7 (38.89%) retained chromosomal amplifications and 2 (11.11%) lost amplifications entirely. For the 76 primary tumors without any amplifications, most PDX models (64.47%) remained unamplified, although some gained chromosomal (22.37%) or ecDNA (13.16%) amplifications.

To analyze potential technical causes of differences in ecDNA status between PDX models and human tumors, we visually inspected WGS coverage profiles at the genomic ecDNA loci. First, we examined the three PDX models for which no ecDNA was detected, even though the human tumors from which they were derived were ecDNA-positive. In two of the three PDX models, increased WGS coverage was observed at the ecDNA loci, suggesting that ecDNA amplifications may be present in the PDX models but remained below the detectable sequencing coverage threshold (Additional file 4: Fig. S1). We next evaluated the 19 cases that exhibited de novo ecDNA emergence in the PDX models that were not observed in the corresponding human tumor. Interestingly, 13 of the 19 (68.42%) PDX models with newly acquired ecDNA were either NBL (*n* = 4) or OST (*n* = 9), despite these tumor types comprising a smaller proportion (38/127, 29.92%) of the cohort with paired samples analyzed in this study (Additional file 5: Table S4). One of the four NBL PDX models that acquired ecDNA-*MYCN* amplifications had a chromosomal *MYCN* amplification in the human tumor (Additional file 4: Fig. S2a), whereas the other three did not have visible traces of WGS enrichment in the human tumors at the *MYCN* locus (Additional file 4: Fig. S2b–d). Seven of the nine OST PDX models that gained ecDNA showed chromosomal amplifications in the human tumors, whereas the other two models did not show any traces of WGS enrichment (Additional file 4: Fig. S2e). While more research is required, these observations suggest that the larger number of ecDNA-positive PDX tumors compared to human tumors as observed for some tumor types (Fig. 2a) might be caused by de novo emergence, or by the outgrowth of an ecDNA + clone undetectable in the available data of the human tumor, during the development of PDX models.

Further stratification by tumor type provided additional context into differences in ecDNA status comparing human tumors and PDX models (Fig. 2b). RMS and RBL were the only tumor types in which ecDNA was lost in the PDX models, whereas ecDNA gains were observed in multiple cancer types, most prominently NBL and OST. Chromosomal amplification gains occurred in HGG, HGS, MBL, NBL, OST, and RMS, while chromosomal losses were observed in OS and RBL. Across cancer types, primary tumors without amplifications predominantly remained unamplified in PDX models (49/76, 64.47%; Fig. 2b). Overall, these cancer type-specific patterns demonstrate that while focal amplifications are generally maintained in PDX models, certain tumor types may display a tendency to gain or lose amplifications during engraftment.

To assess whether specific oncogenes are preferentially retained during PDX engraftment, we evaluated gene-level ecDNA retention across all tumor–PDX pairs using Fisher’s exact tests. Among the 87 ecDNA-amplified oncogenes identified, 34 were present in at least two tumor–PDX pairs and were therefore included in statistical analyses. Although all 34 oncogenes exhibited complete (100%) retention with no instances of loss, the strength of statistical significance differed across genes simply because some occurred in more tumor–PDX pairs, providing greater statistical power. For example, *MYCN* showed the strongest signal (12/12 retained; FDR = 0.00015, Fig. 2c), while *MYC*, *MDM2*, *CCNE1*, and *CDK4* also demonstrated perfect retention with FDR < 0.10. These findings indicate that ecDNA-amplified oncogenes, particularly those involved in cell-cycle regulation (*MYCN*, *CCNE1*) [47–49], apoptosis (*MDM2*) [50], and transcriptional control (*MYC*, *PAX7*) [51, 52], are consistently and universally preserved during PDX engraftment.

We next examined whether genes co-amplified with *MYC* or *MYCN* on ecDNA are coordinately retained or independently lost in PDX models (Fig. 2d). This analysis was restricted to 11 tumor-PDX pairs in which ecDNA amplifications were confirmed in both the primary tumor and PDX and were *MYC*- or *MYCN*-amplified. Among these 11 pairs, we identified 17 genes co-amplified with these oncogenes. *MYCN* exhibited 100% retention (7/7), while *MYC* was retained in 75% (3/4) of cases. *PVT1*, a well-characterized long non-coding RNA near the *MYC* gene locus, was retained in 2/3 cases (67%). While some co-amplified genes, such as *PAX7* and *TRIB1* were retained together with *MYC* or *MYCN* in the *PDX* models, other genes were lost from ecDNA in PDX models, including *MDM4*, *FGFR4*, and *CNTN2*. Interestingly, some genes were gained on *MYC*/*MYCN*-amplified ecDNA in PDX models but were absent in the primary tumor, including *TERT* and *CTNND2*. These findings suggest that while the core *MYC*/*MYCN*-containing amplicons are highly stable during PDX engraftment, the composition of co-amplified genes is prone to dynamic changes, with certain passengers being gained or lost independently of the primary oncogenic driver. However, given that many of these co-amplified genes appear in only isolated cases, the extent to which these observations can be generalized is limited.

**Fig. 3:**
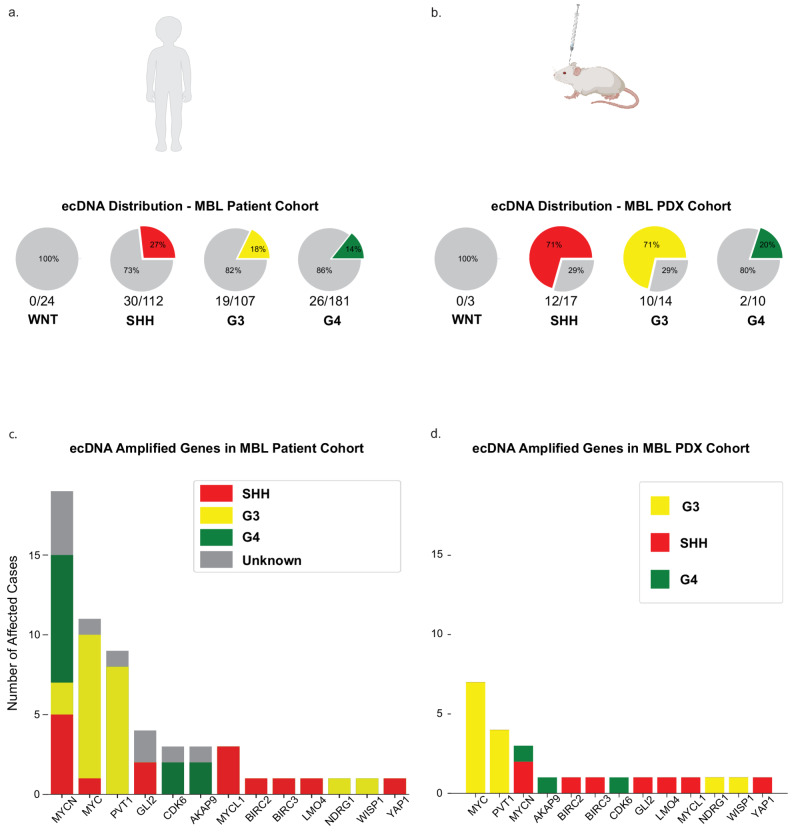
Frequency of ecDNA and ecDNA-amplified genes in human tumors and PDX models across medulloblastoma subgroups. **a** Frequency of ecDNA in the MBL patient cohort grouped by molecular subgroup. The distribution of ecDNA-positive cases across molecular subgroups was as follows: WNT (0/24), SHH (30/112), G3 (19/107), and G4 (26/181). **b** Frequency of ecDNA in the MBL PDX model cohort grouped by molecular subgroup. The distribution was: WNT (0/3), SHH (12/17), G3 (9/14), and G4 (2/10). **c** Frequency of ecDNA-amplified genes in the MBL patient cohort grouped by molecular subgroup. **d** Frequency of ecDNA-amplified genes in the MBL PDX model cohort grouped by molecular subgroup

Most tumor types can be stratified into molecular subgroups with distinct clinical and molecular characteristics. For example, in a previous study of 468 MBL patients [3], we observed a varying frequency of ecDNA in the different MBL subgroups where ecDNA was detected in 27% of SHH, 18% of Group 3, and 14% of Group 4 MBL tumors, while being absent in WNT tumors (Fig. 3a) [3]. While the available cohort of PDX models is not large enough for a representative stratification of all childhood tumor types into their molecular subgroups, there is a sufficiently large number of 46 MBL PDX models representing the four molecular MBL subgroups. As in human tumors, no ecDNA was observed in MBL WNT PDX models, while a substantial amount of SHH (71%), Group 3 (71%), and Group 4 (20%) MBL PDX models contained ecDNA (*N* = 44, Fig. 3b). Thus, the enrichment of ecDNA in MBL PDX models compared to human MBL patients was concentrated on PDX models of the SHH MBL and Group 3 MBL subgroups (Fisher’s exact test, *p* < 0.002 for both subgroups). When comparing genes amplified on ecDNA in human MBL tumors and MBL PDX models, we observe 13 oncogenes consistently amplified across both PDX models and patient tumors, including *MYC* and *MYCN* (Fig. 3c, d).

### ecDNA sequence is largely conserved in PDX models

**Fig. 4:**
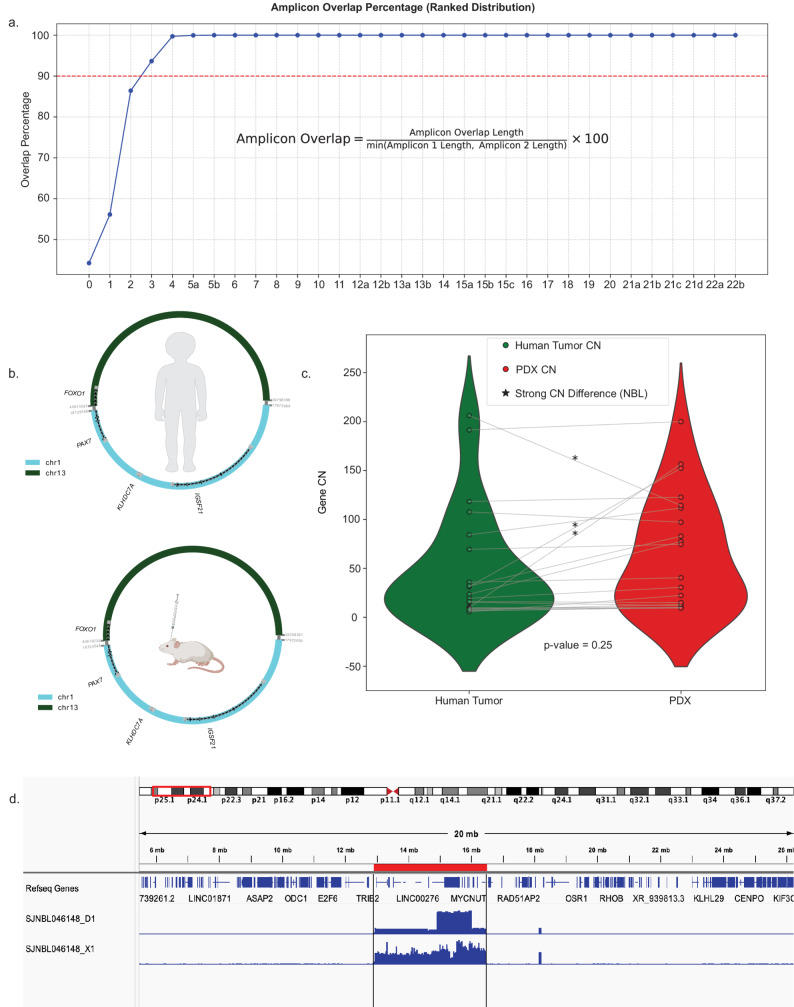
Amplicon and copy number similarity of ecDNA-positive human tumors and their PDX models. **a** Ranked line chart of amplicon length coverage percentage between ecDNA-positive amplicon amplicons in human tumor-PDX model pairs. Red line cutoff for pairs with amplicon overlap coverage at 90%. **b** Circos circular visualizations of patient SJRHB010468 named SJRHB010468_D1 and SJRHB010468_X1, respectively. **c** Paired copy number comparison violin plot after removing pairs with low amplicon length coverage percentage. Contains 23 human tumors and 23 PDX models and 24 paired ecDNAs. Removed samples of patients SJNBL013763, SJRHB011, SJOS001126, SJOS013768, SJOS030605, SJRHB063823, and Med1911FH. **d** Amplicon graphs of NBL pair (SJNBL046148) with a decrease of ecDNA-amplified oncogene copy number in the PDX sample. HT indicates human tumor and PDX indicates PDX sample. Red horizontal line spanning ecDNA-positive region

To assess whether ecDNA sequences are conserved in PDX models compared to their tumors of origin, we employed a metric derived from Jaccard similarity [11, 13] to calculate DNA sequence similarities of ecDNA amplifications (see Methods). We identified 45 ecDNA sequences from 30 tumor-PDX pairs in which both the human tumors and the PDX models were ecDNA-positive, allowing a direct comparison of ecDNA sequence similarities. (Additional file 6: Table S5). Among the 30 ecDNA-positive human tumor-PDX pairs, four (13%) showed no overlap in amplicon coverage, indicating that ecDNA amplifications arose from entirely different genomic loci in the tumor and PDX models. The lack of overlap suggests that, in these cases, the PDX models may have developed from tumor clones harboring distinct ecDNAs, rather than directly mirroring the ecDNA architecture of the original tumor. In the remaining 26 human tumor-PDX pairs, we observed 38/45 (84.4%) ecDNAs originating from near-identical genomic loci in the human tumor and PDX model (amplicon overlap > 0.99; Fig. 4a) and 4/45 (8.89%) ecDNAs that only partially overlapped (amplicon overlap < 95%).

We next evaluated amplicon similarity scores that quantify shared features between focal amplifications based on genomic composition and breakpoint locations. Applying this approach to the 26 tumor-PDX pairs with overlapping ecDNAs, we observed distinct levels of conservation (see Methods). Very high amplicon similarity scores exceeding 0.995 were observed in 6/26 (23%) tumor–PDX pairs (Additional file 4: Fig. S3a). Two tumor-PDX pairs exhibited complete sequence conservation (similarity score = 1.0): an ARMS case (SJRHB010468) with conserved amplification of *FOXO1*, *IGSF21*, *KLHDC7A*, and *PAX7* on chromosome 13 (Fig. 4b), and an OST case (SJOS016016) with preserved amplicons on chromosome 1, including *MCL1*, *ARNT*, *MLLT11*, and *SETDB1* (Additional file 4: Fig. S4a). However, the range of similarity scores was between 0.11–1 with a mean of 0.66 (Additional file 4: Fig. S3a). These lower similarity scores are largely driven by lower segment and breakpoint scores averaging at 0.87 and 0.58, respectively (Additional file 4: Fig. S3b, c). Examples of moderate to high conservation included an OST pair (SJOS001121) with a similarity score of 0.84 (Additional file 4: Fig. S4b), and an NBL case (SJNBL124) with a score of 0.74, despite breakpoint variability (Additional file 4: Fig. S4c). A divergent RMS case (SJRHB071775) showed minimal structural similarity (score 0.35), largely due to low breakpoint concordance (0.057) (Additional file 4: Fig. S4d). Overall, these findings demonstrate that the majority of ecDNA-amplified sequences are conserved at the sequence level, with structural variability arising predominantly from breakpoint divergence, or due to technical uncertainties of breakpoint reconstructions, rather than wholesale loss of amplified content.

### PDX models recapitulate the ecDNA copy number observed in human tumors

We next investigated whether the copy number of (onco-) genes amplified on ecDNA is conserved in PDX models compared to the human tumors from which they are derived. Here, we examined 24 ecDNAs from 23 primary tumors that have ecDNA-amplified genes in both human primary tumors and PDX models and whose ecDNAs have an amplicon overlap coverage of = 90%. While the average ecDNA copy number was higher in the PDX models than in their matched primary tumors, this difference was not statistically significant (Wilcoxon signed-rank test, *p* = 0.25; Fig. 4c). Overall, ecDNA copy numbers appeared to be relatively stable between human tumors and PDX models, with a few notable outliers. For example, two NBL tumors (SJNBL013762 and SJNBL030824) showed pronounced increases in ecDNA copy number in the PDX and both harbored *MYCN*. Conversely, one *MYCN*-amplified tumor (SJNBL046148) showed a decrease in ecDNA copy number in the PDX (Fig. 4c). Visual inspection of the *MYCN* gene locus shows that the reduction of *MYCN* copy number is constrained to the loss of a narrow hyper-amplification centered at the *MYCN* gene (Fig. 4d) which is embedded in a larger ecDNA amplification conserved in both the human tumor and PDX model. Overall, these findings demonstrate broad ecDNA copy number conservation in PDX tumors across pediatric cancers; however, individual cases linked to *MYCN* show significant changes in oncogene copy number.

### ecDNA-positive cells exhibit distinct clonal dynamics during PDX development

We have previously employed multiome (ATAC/RNA) single nucleus sequencing to identify and characterize tumor cells harboring ecDNA in a primary SHH MBL tumor (‘RCMB56-ht’) [3]. To analyze the clonal behavior of ecDNA-positive tumor cells during PDX development, we now profiled a PDX tumor derived from RCMB56-ht (i.e. RCMB56-pdx) using the same multiome single nucleus sequencing approach. In addition, we used multiome single nucleus sequencing to analyze a primary G3 MBL tumor amplifying *MYC* on ecDNA (i.e. RC123-ht) and a PDX tumor that we derived from that patient's tumor (i.e. RC124-pdx).

**Fig. 5:**
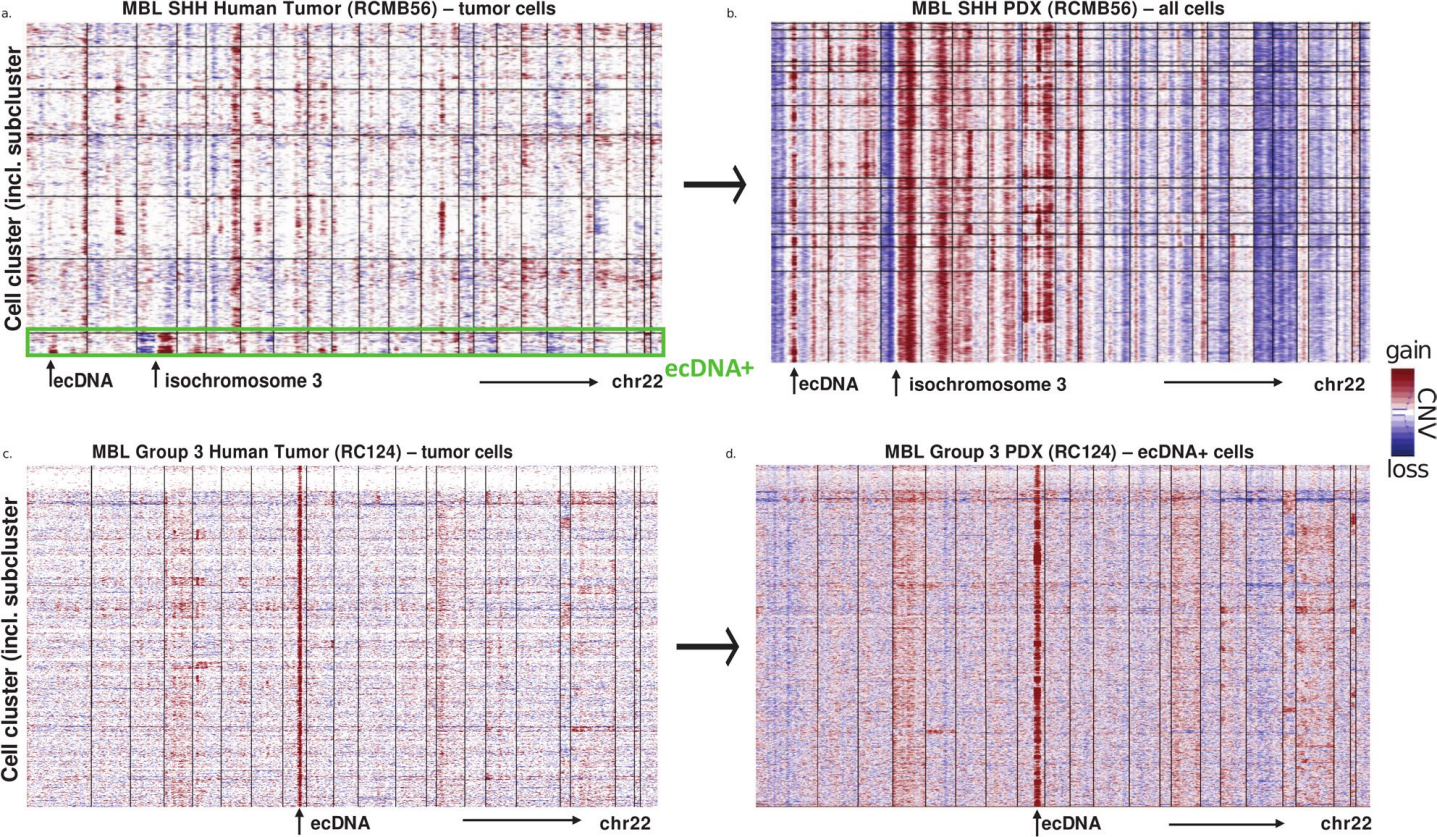
Clonal dynamics of ecDNA-positive tumor cells in MBL tumor-PDX pairs. **a** Copy-number profiles of individual cells in the MBL SHH tumor RCMB56-ht, including the 8% ecDNA + tumor cells clustered at the bottom. **b** Copy-number profiles of the RCMB56-pdx cells, showing that almost all cells in the PDX tumor resemble the CNV profile of the ecDNA + tumor cells in the human tumor. **c** Copy-number profiles of the ecDNA-positive tumor cells of the Group 3 MBL tumor RC124-ht, representing > 99% of all cells in the human tumor. **d** Copy-number profiles of the ecDNA-positive RC124-pdx cells, showing that almost all cells in the PDX tumor resemble the CNV profile of the ecDNA + tumor cells in corresponding human tumor, including the *MYC* amplification on chromosome 8

In the primary SHH MBL tumor sample (RCMB56-ht), we estimated that only 8% of tumor cells contained ecDNA amplifications [3] (Fig. 5a, see Methods). Analysis of the corresponding PDX model (RCMB56-pdx) revealed that almost all cells (99.6%, 9,608 out of 9,620) contained the ecDNA amplifications present in the ecDNA-positive tumor cells of the human tumor (Fig. 5b). Integration and batch correction of single-cell transcriptomes (see Methods [36]) revealed substantial transcriptional similarities between RCMB56-pdx cells and the ecDNA-positive tumor cells in RCMB56-ht (Additional file 4: Fig. S5). The combined similarity of gene expression and copy number profiles suggests that the PDX tumor originated from clonal expansion of the ecDNA-positive cells in the primary tumor. In contrast to the SHH MBL tumor (RCMB56-ht), ecDNA harboring the *MYC* oncogene was present in almost all the individual cells in the primary human G3 MBL tumor (RC124-ht) (Fig. 5c). Single cell analysis showed that the same ecDNA was omnipresent in the corresponding PDX tumor (RC124-pdx, Fig. 5d), demonstrating maintenance of ecDNA throughout PDX development.

Overall, these findings revealed that ecDNA-positive tumor cells can exist either as a minor subpopulation or as a widespread cell population within primary human tumors. In RCMB56, ecDNA-bearing cells were initially rare but underwent marked clonal expansion during PDX development, suggesting a strong selective advantage for ecDNA-driven clones. Conversely, in RC124, ecDNA-positive cells were already abundant in the primary tumor and persisted as the dominant clone in the PDX. Despite these differences in initial prevalence, the resulting PDX tumors in both cases were composed almost entirely of ecDNA-positive cells. This convergence highlights the potent growth advantage conferred by ecDNA and suggests that ecDNA-bearing clones are preferentially selected during PDX establishment, regardless of their frequency in the original tumor.

## Discussion

This study provides a comprehensive overview of ecDNA in pediatric cancer PDX models, highlighting its prevalence, oncogenic content, and conservation relative to patient tumors. We observed that ecDNA frequently recapitulates oncogene amplifications found in human cancers, is generally preserved during PDX establishment, and reflects subtype-specific patterns across tumor types. These findings support the utility of PDX models in studying ecDNA biology and their implications for pediatric cancer progression and treatment.

The observation that ecDNA is enriched in PDX models compared to primary tumors suggests a selective advantage for ecDNA-positive cells during xenograft establishment. This was particularly evident in neuroblastoma and medulloblastoma, where significant enrichment of ecDNA was observed in the PDX models of these tumor types. The preferential outgrowth of ecDNA-positive cells, as demonstrated in our SHH-MBL RCMB56 case study in which an ecDNA-positive subclone (8% of cells) expanded to dominate the PDX model (99.6% of cells), highlights the aggressive and proliferative nature of ecDNA-harboring cells. This expansion likely reflects a selective pressure during PDX establishment, potentially favoring cells with enhanced proliferative capacity conferred by ecDNA-amplified oncogenes.

Conservation of ecDNA sequences and amplification of oncogenes between primary tumors and PDX models is encouraging for translational cancer research. With approximately 84% of ecDNA amplicons displaying substantial sequence conservation (> 90% overlap coverage), PDX models largely recapitulated the ecDNA sequences amplified in primary tumors. However, the variability of the breakpoint positions and the differences in the overall similarity scores of the amplicons indicate recombination of ecDNA during model development, although reconstruction error of ecDNA from WGS data may also play a role. These factors contributing to ecDNA dynamics should be considered when using PDX models to investigate patient tumor behavior, particularly in cases like *MYCN*-amplified neuroblastoma where substantial enrichment of ecDNA occurs. In such cases, future studies should account for elevated ecDNA dosage via normalization analysis with ecDNA copy number and incorporation of parallel comparisons with patient-matched samples to provide context for observed differences in gene expression, growth kinetics, or therapeutic response. In addition, differences in ecDNA abundance across tumor-PDX pairs may result in altered sensitivity or resistance to pharmacological or genetic inhibition of ecDNA-amplified oncogenes, potentially indicating variations in dosage and heterogeneity that influence tumor behavior. Integrating quantitative ecDNA profiling and functional assays for primary tumor-PDX pairs will enable more accurate inferences of tumor behavior and therapeutic response that reflect patient context.

While there was a trend towards increased copy numbers of ecDNA-amplified oncogenes in PDX models compared to their human tumors of origin, this difference did not reach statistical significance, indicating that ecDNA copy numbers are generally conserved during PDX model development. The particularly dramatic copy number increases in *MYCN*-amplified neuroblastoma cases underscores the potential importance of this oncogene during PDX establishment.

The emergence of ecDNA-positive PDX tumors derived from some ecDNA-negative human tumors has raised important considerations for model interpretation. This phenomenon was particularly prevalent in neuroblastomas and osteosarcomas, suggesting that tumor-specific mechanisms influence ecDNA formation selection or stability. Whether these newly detected ecDNAs represent outgrowth of pre-existing minor subclones below detection thresholds in primary samples, or truly de novo formation during PDX establishment, remains an important question for future investigation.

Future studies should investigate the mechanisms driving the selective advantage of ecDNA-positive cells during PDX establishment and explore whether similar enrichment occurs in other model systems, such as organoids or cell lines. Understanding the factors that promote ecDNA formation and maintenance in different environmental contexts could reveal new therapeutic vulnerabilities.

In conclusion, our findings validate PDX models as valuable tools for studying ecDNA biology in childhood cancers. Longitudinal sampling during PDX tumor growth and under therapeutic pressure could provide valuable insights into the dynamics of molecular evolution, clonal selection, and ecDNA-driven therapy resistance.

## Conclusions

Our study provides a comprehensive analysis of ecDNA in pediatric cancer PDX models. We demonstrate that ecDNA status, amplified oncogenic content, sequence composition are predominantly conserved in PDX models relative to their primary tumors of origin, validating their utility as preclinical tools for studying ecDNA biology. Despite variability in breakpoint concordance and occasional ecDNA enrichment in PDX models, the core oncogenic amplifications driving primary tumor growth and proliferation are consistently preserved. The favored expansion of ecDNA-positive clones during PDX establishment further reinforces the potent selective advantage conferred by ecDNA in vivo. These findings serve as a foundation for leveraging PDX models in longitudinal studies of ecDNA evolution under therapeutic pressure, potentially offering new insights into ecDNA-associated treatment resistance and novel therapeutic targets in pediatric oncology.

## Supplementary Information

Additional file 1: Table S1. Sample information of St. Jude PDX models and Rady PDX models.

Additional file 2: Table S2. AmpliconClassifier results of St. Jude PDX models and paired human tumors.

Additional file 3: Table S3. Paired ecDNA status results of PDX models and human tumors of origin.

Additional file 4: Figures S1–S5. Supplementary figures including IGV visualizations of ecDNA genomic regions, amplicon similarity score distributions, amplicon graphs of ecDNA-positive tumor-PDX pairs, and single-cell clustering of the RCMB56 tumor-PDX pair.

Additional file 5: Table S4. Paired tumors with ecDNA gains in PDX models and corresponding genes.

Additional file 6: Table S5. Amplicon similarity results of ecDNA-positive human tumor-PDX pairs.

## Data Availability

Research data supporting this publication are available from the St. Jude Cloud Genomics Platform repository located at https://platform.stjude.cloud/data/samples [9]. The dataset containing the results for these samples is now available through an interactive web portal at https://ccdi-ecdna.org/pdx [5, 53]. Source code for software applets used to run AmpliconSuite-pipeline on the DNANexus is available at: https://github.com/chavez-lab/ampliconsuite-dnanexus-applet [15]. Source code to regenerate analyses and figures is available at https://github.com/rishaan1/ecDNA-PDX-Study/tree/main/scripts [54].

## Abbreviations

ecDNA: Extrachromosomal DNA
PDX: Patient-derived xenograft
WGS: Whole-genome sequencing
MBL: Medulloblastoma
OST: Osteosarcoma
NBL: Neuroblastoma
RMS: Rhabdomyosarcoma
RBL: Retinoblastoma
HGG: High-Grade Glioma
WT: Wilms’ Tumor
ES: Ewing Sarcoma
DSCRT: Desmoplastic Small Round Cell Tumor
HGS: High-Grade Sarcoma
ARMS: Alveolar Rhabdomyosarcoma
CBTN: Children’s Brain Tumor Network
CNV: Copy number variation
DEG: Differentially expressed gene
ATAC: Assay for Transposase-Accessible Chromatin
FDR: False discovery rate
FPKM: Fragments per kilobase million
G3: Group 3
G4: Group 4
ICGC: International Cancer Genome Consortium
IGV: Integrative Genomics Viewer
NSG: NOD-SCID *IL2Rg* null
scATAC-seq: Single-cell assay for transposase-accessible chromatin sequencing
scRNA-seq: Single-cell RNA sequencing
SHH: Sonic Hedgehog
SV: Structural variant
