# Supplementary material for: Preservation and clonal behavior of extrachromosomal DNA in patient-derived xenograft models of childhood cancers
Source: Genome Med. 2026 May 28;18:110. doi: 10.1186/s13073-026-01676-0 (PMC13403850; doi:10.1186/s13073-026-01676-0)
Supplement: Supplementary file 4 — Additional file 4: Figures S1–S5. Supplementary figures including IGV visualizations of ecDNA genomic regions, amplicon similarity score distributions, amplicon graphs of ecDNA-positive tumor-PDX pairs, and single-cell clustering of the RCMB56 tumor-PDX pair. [file 13073_2026_1676_MOESM4_ESM.pdf]

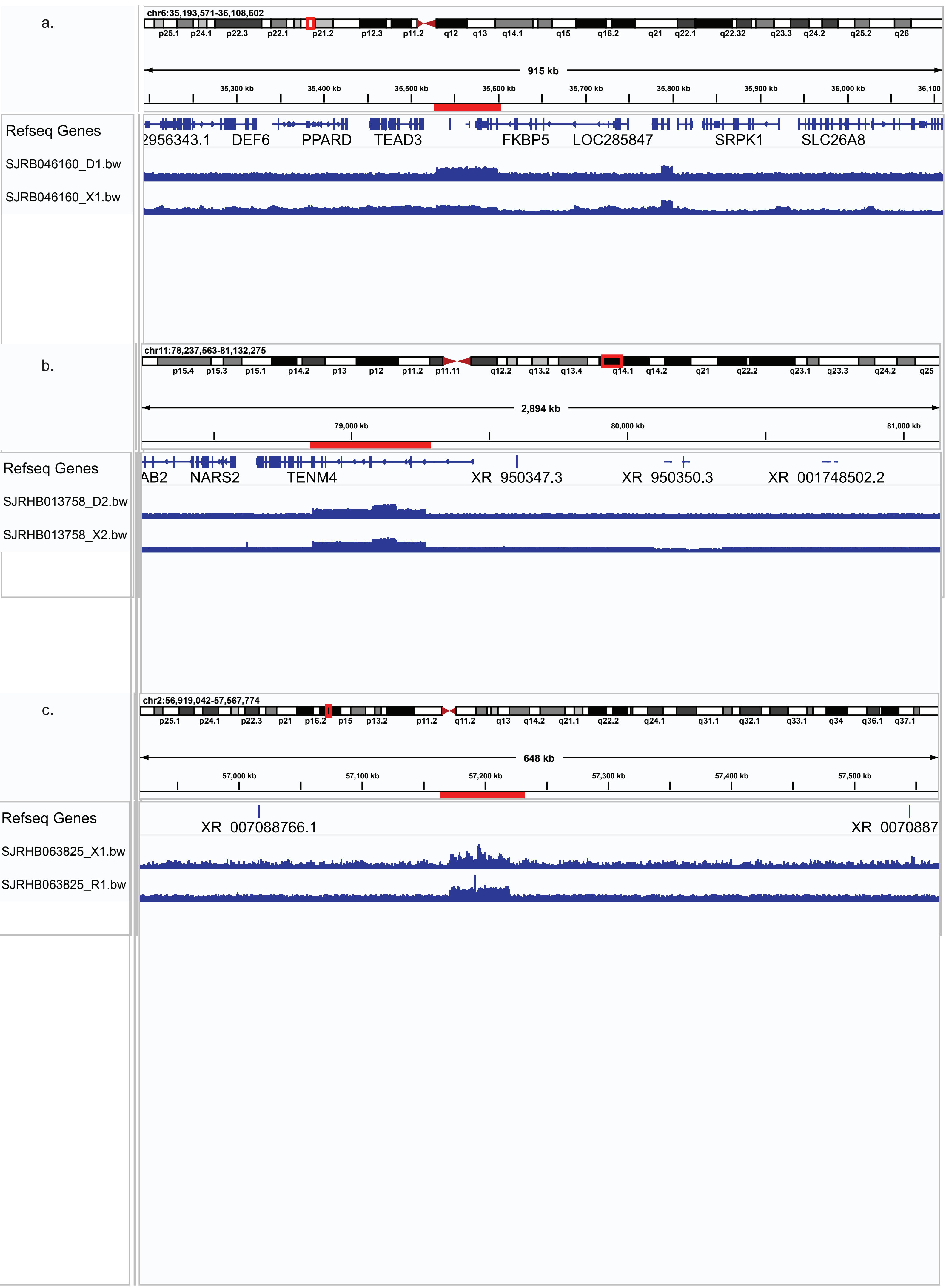
a.
chr6:35,193,571-36,108,602
p25.1 p24.1 p22.3 p22.1 p21.2 p12.3 p11.2 q12 q13 q14.1 q15 q16.2 q21 q22.1 q22.32 q23.3 q24.2 q25.2 q26
915 kb
35,300 kb 35,400 kb 35,500 kb 35,600 kb 35,700 kb 35,800 kb 35,900 kb 36,000 kb 36,100
Refseq Genes
2956343.1 DEF6 PPARD TEAD3 FKBP5 LOC285847 SRPK1 SLC26A8
SJRB046160_D1.bw
SJRB046160_X1.bw
b.
chr11:78,237,563-81,132,275
p15.4 p15.3 p15.1 p14.2 p13 p12 p11.2 p11.11 q12.2 q13.2 q13.4 q14.1 q14.2 q21 q22.2 q23.1 q23.3 q24.2 q25
2,894 kb
79,000 kb 80,000 kb 81,000 kb
Refseq Genes
AB2 NARS2 TENM4 XR 950347.3 XR 950350.3 XR 001748502.2
SJRHB013758_D2.bw
SJRHB013758_X2.bw
c.
chr2:56,919,042-57,567,774
p25.1 p24.1 p22.3 p21 p16.2 p15 p13.2 p11.2 q11.2 q13 q14.2 q21.1 q22.2 q24.1 q31.1 q32.1 q33.1 q34 q36.1 q37.1
648 kb
57,000 kb 57,100 kb 57,200 kb 57,300 kb 57,400 kb 57,500 kb
Refseq Genes
XR 007088766.1 XR 0070887
SJRHB063825_X1.bw
SJRHB063825_R1.bw

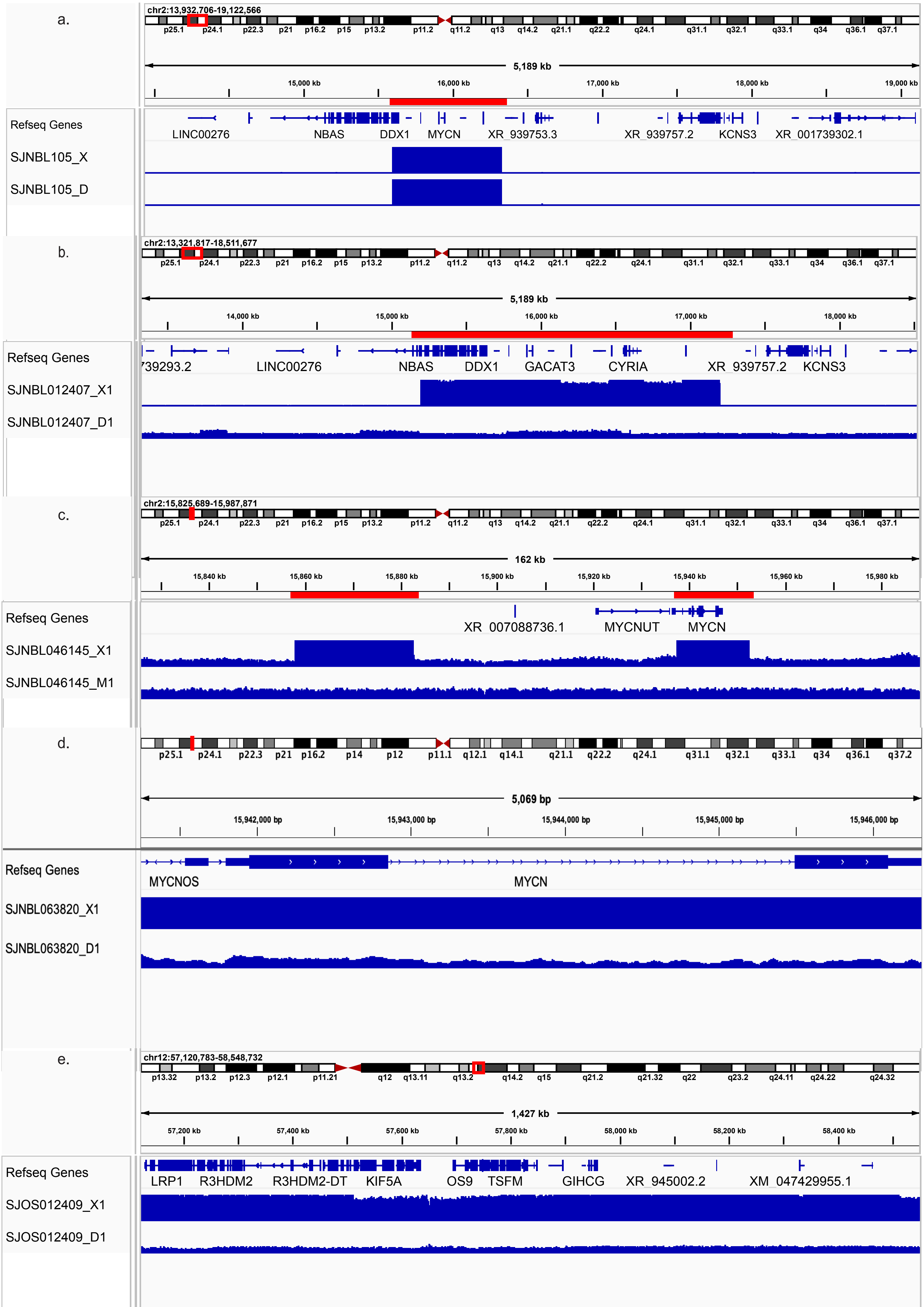
a.
chr2:13,932,706-19,122,566
5,189 kb
Refseq Genes
LINC00276
NBAS
DDX1
MYCN
XR_939753.3
XR_939757.2
KCNS3
XR_001739302.1
SJNBL105_X
SJNBL105_D
b.
chr2:13,321,817-18,511,677
5,189 kb
Refseq Genes
LINC00276
NBAS
DDX1
GACAT3
CYRIA
XR_939757.2
KCNS3
SJNBL012407_X1
SJNBL012407_D1
c.
chr2:15,825,689-15,987,871
162 kb
Refseq Genes
XR_007088736.1
MYCNUT
MYCN
SJNBL046145_X1
SJNBL046145_M1
d.
5,069 bp
Refseq Genes
MYCNOS
MYCN
SJNBL063820_X1
SJNBL063820_D1
e.
chr12:57,120,783-58,548,732
1,427 kb
Refseq Genes
LRP1
R3HDM2
R3HDM2-DT
KIF5A
OS9
TSFM
GIHCG
XR_945002.2
XM_047429955.1
SJOS012409_X1
SJOS012409_D1

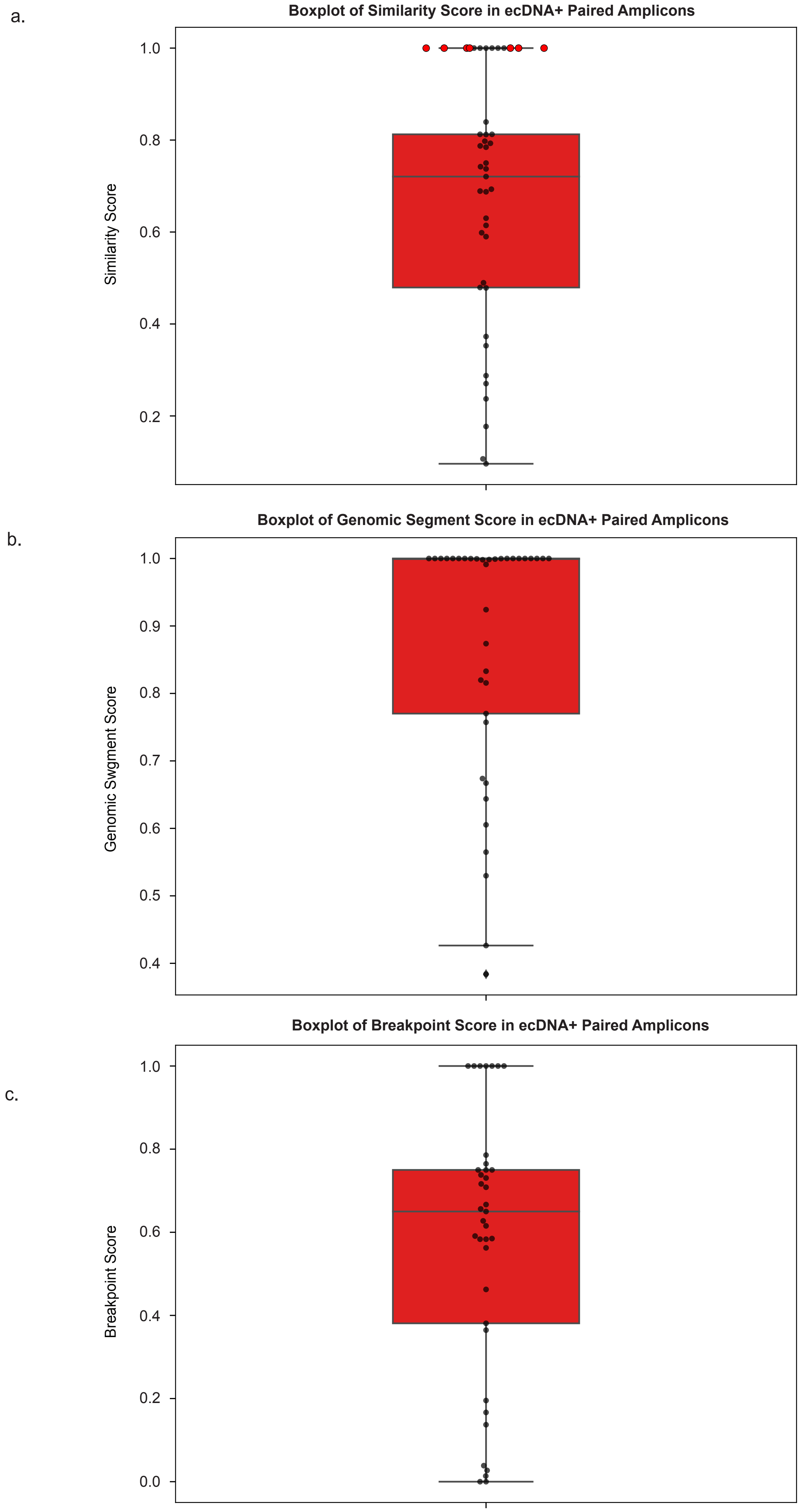

a.
Boxplot of Similarity Score in ecDNA+ Paired Amplicons
Similarity Score
1.0
0.8
0.6
0.4
0.2
b.
Boxplot of Genomic Segment Score in ecDNA+ Paired Amplicons
Genomic Swgment Score
1.0
0.9
0.8
0.7
0.6
0.5
0.4
c.
Boxplot of Breakpoint Score in ecDNA+ Paired Amplicons
Breakpoint Score
1.0
0.8
0.6
0.4
0.2
0.0

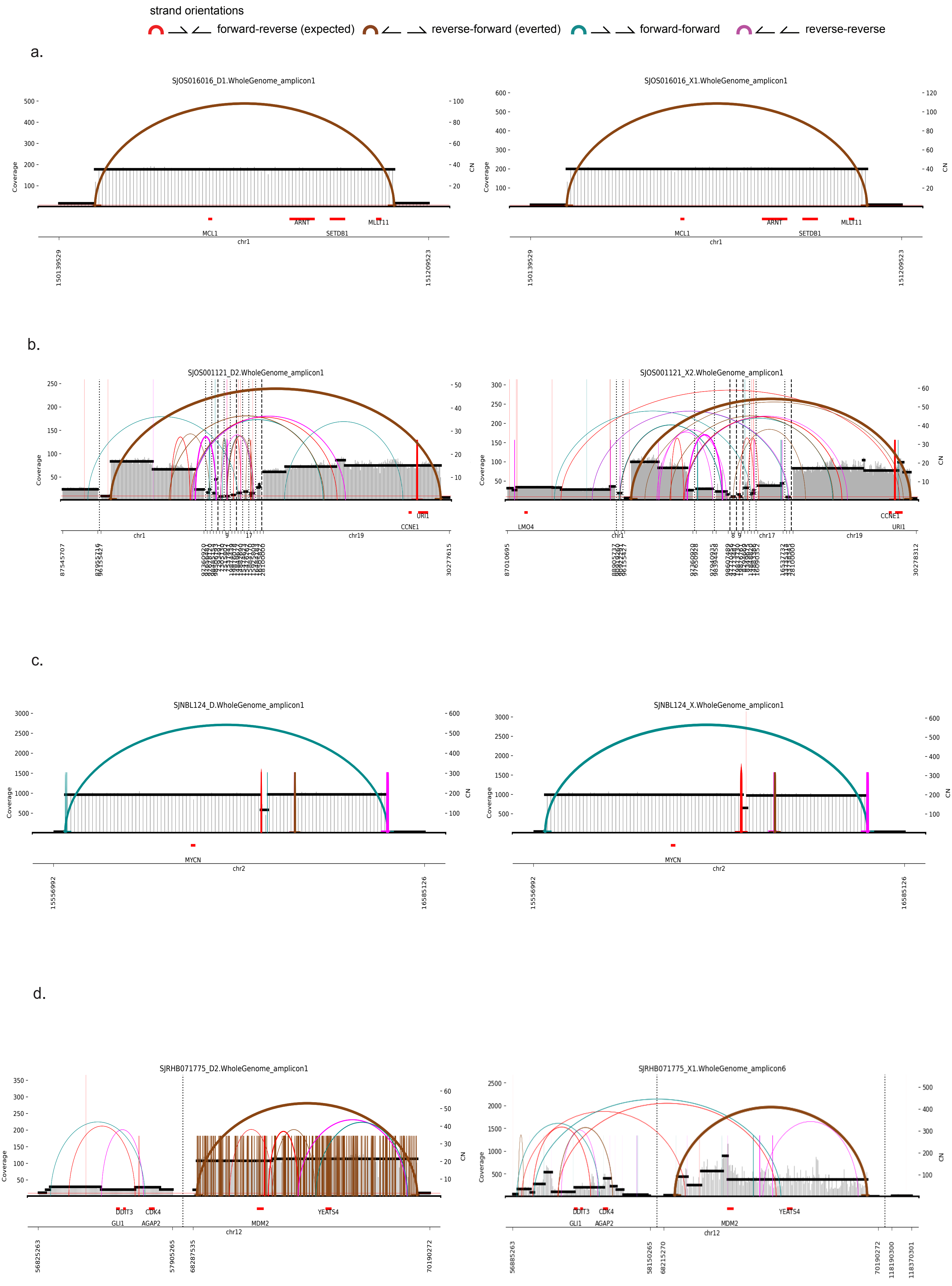
strand orientations
forward-reverse (expected)
reverse-forward (everted)
forward-forward
reverse-reverse
a.
SJOS016016_D1.WholeGenome_amplicon1
SJOS016016_X1.WholeGenome_amplicon1
Coverage
CN
MCL1
ARNT
SETDB1
MLLT11
chr1
150139529
151209523
b.
SJOS001121_D2.WholeGenome_amplicon1
SJOS001121_X2.WholeGenome_amplicon1
LMO4
CCNE1
URI1
chr1
chr17
chr19
87545707
30277615
87010695
30278312
c.
SJNBL124_D.WholeGenome_amplicon1
SJNBL124_X.WholeGenome_amplicon1
MYCN
chr2
15556992
16585126
d.
SJRHB071775_D2.WholeGenome_amplicon1
SJRHB071775_X1.WholeGenome_amplicon6
DDIT3
CDK4
GLI1
AGAP2
MDM2
YEATS4
chr12
5825263
57905265
68287535
70190272
5685263
58150265
68215270
118190300
118370301

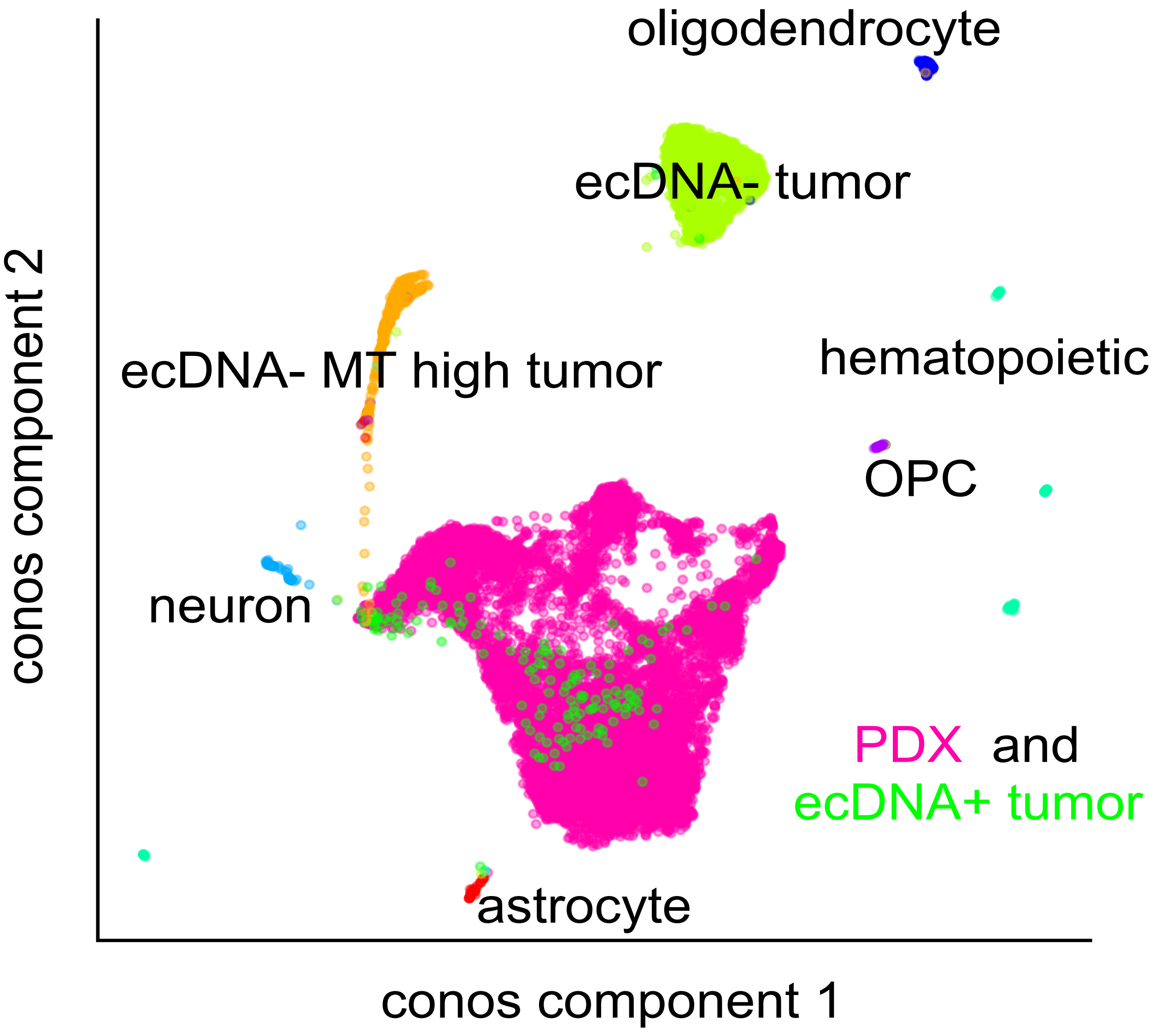
oligodendrocyte
ecDNA- tumor
ecDNA- MT high tumor
hematopoietic
OPC
neuron
PDX and
ecDNA+ tumor
astrocyte
conos component 2
conos component 1

Fig. S1: Visualizations of ecDNA genomic regions amongst human tumorPDX pairs that transitioned from ecDNA-positive to ecDNA-negative status.

Integrative Genomics Viewer (IGV) visualizations of the three tumor-PDX pairs for which ecDNA was lost. Regions with high coverage indicate potential ecDNA locations. The red horizontal line indicates predicted ecDNA-amplified location. _X indicates PDX model and any other annotation indicates human tumor. ecDNA-amplified gene *FKBP5* in patient **(a)** SJRB046160; no ecDNA-amplified genes in the other two primary tumors for patients **(b)** SJRHB013758 and **(c)** SJRHB063825.

Fig. S2: Visualizations of ecDNA genomic regions amongst human tumorPDX pairs that transitioned from ecDNA-negative to ecDNA-positive status.

Integrative Genomics Viewer (IGV) visualizations of the three tumor-PDX pairs for which ecDNA was lost. Regions of high coverage indicate potential ecDNA locations. Red horizontal line indicates predicted ecDNA-amplified location. _X indicates PDX model and any other annotation indicates human tumor. **(a)** Patient SJNBL105 (neuroblastoma), **(b)** Patient SJNBL012407 (neuroblastoma), **(c)** Patient SJNBL046145 (neuroblastoma), **(d)** Patient SJNBL063820 (neuroblastoma), **(e)** Patient SJOS012409 (osteosarcoma).

Fig. S3: Amplicon similarity score metric distribution.

(a) Boxplot showing amplicon similarity score distribution of ecDNA-positive tumor-PDX pairs.
(b) Boxplot showing genomic segment score distribution of ecDNA-positive tumor-PDX pairs. **(c)** Boxplot showing breakpoint sharing score distribution of ecDNA-positive tumor-PDX pairs.

Fig. S4: Amplicon graphs of ecDNA-positive human tumor-PDX pairs.

(a) Osteosarcoma pair (SJOS016016) demonstrating perfect sequence conservation with similarity score 1.0. **(b)** Osteosarcoma pair (SJOS001121) with amplicon similarity score 0.84. **(c)** Neuroblastoma pair (SJNBL124) with amplicon similarity score 0.74. **(d)** Rhabdomyosarcoma pair (SJRHB071775) with amplicon similarity score 0.35.

Fig. S5: Joint single-cell clustering of RCMB56 tumor-PDX pair

Conos clustering of the transcriptome profile in RCMB56-PDX, overlaid on the transcriptome profile of RCMB56-HT to highlight similarities and differences in gene expression patterns between the two samples.
